# Supplementary figures and images for: Timing of host feeding drives rhythms in parasite replication
Source: PLoS Pathog. 2018 Feb 26;14(2):e1006900. doi: 10.1371/journal.ppat.1006900 (PMC5843352; doi:10.1371/journal.ppat.1006900)

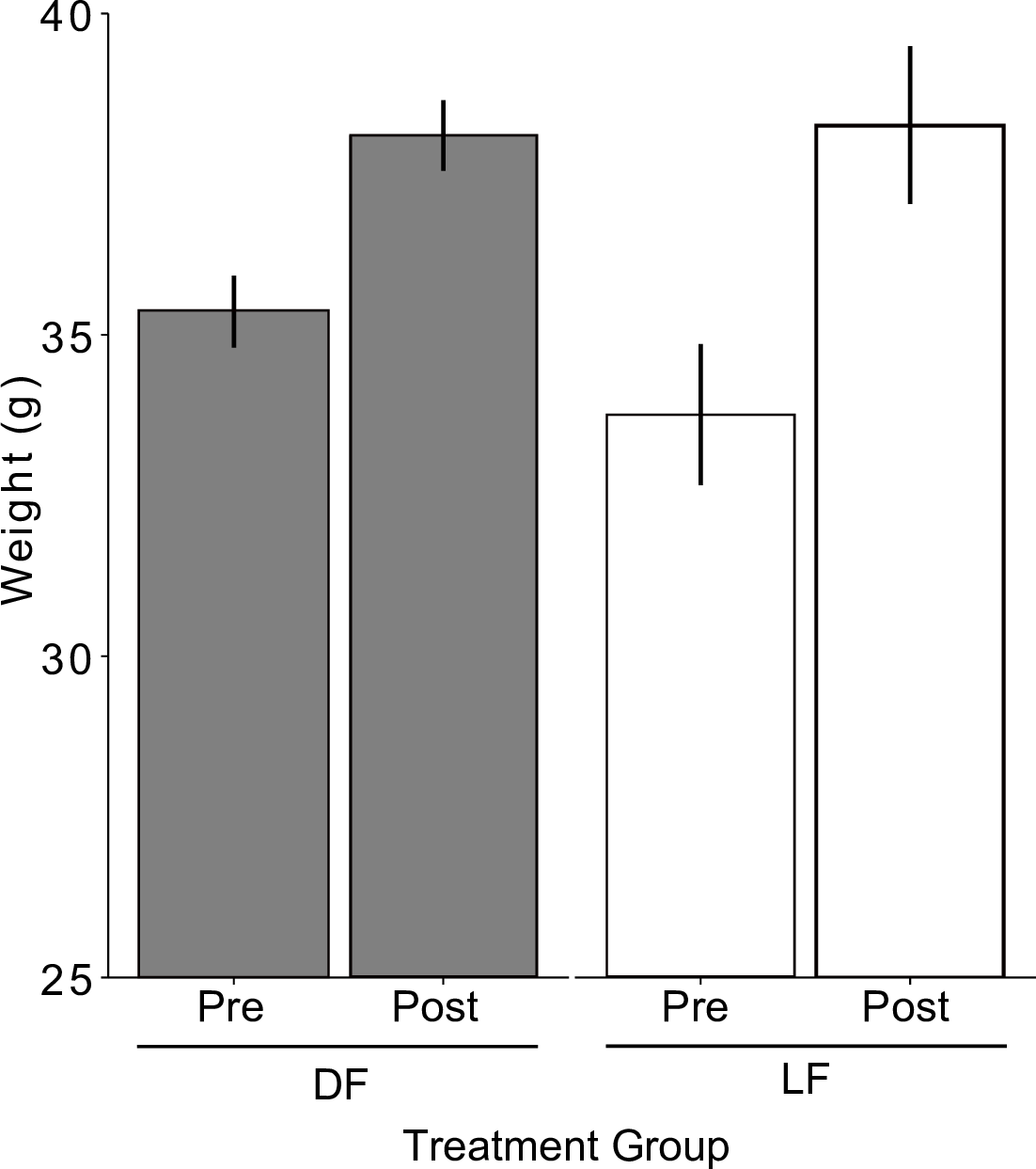

Supplement: S1 Fig — Mean ± SEM body weight for N = 10 mice per treatment group. All mice were weighed before feeding time manipulation (pre) and after recovering from infections (post). All mice gained weight over the 4 weeks of the experiment, and weight did not differ significantly at either point between light fed (LF) and dark fed (DF) mice. (TIF) [file ppat.1006900.s001.tif]

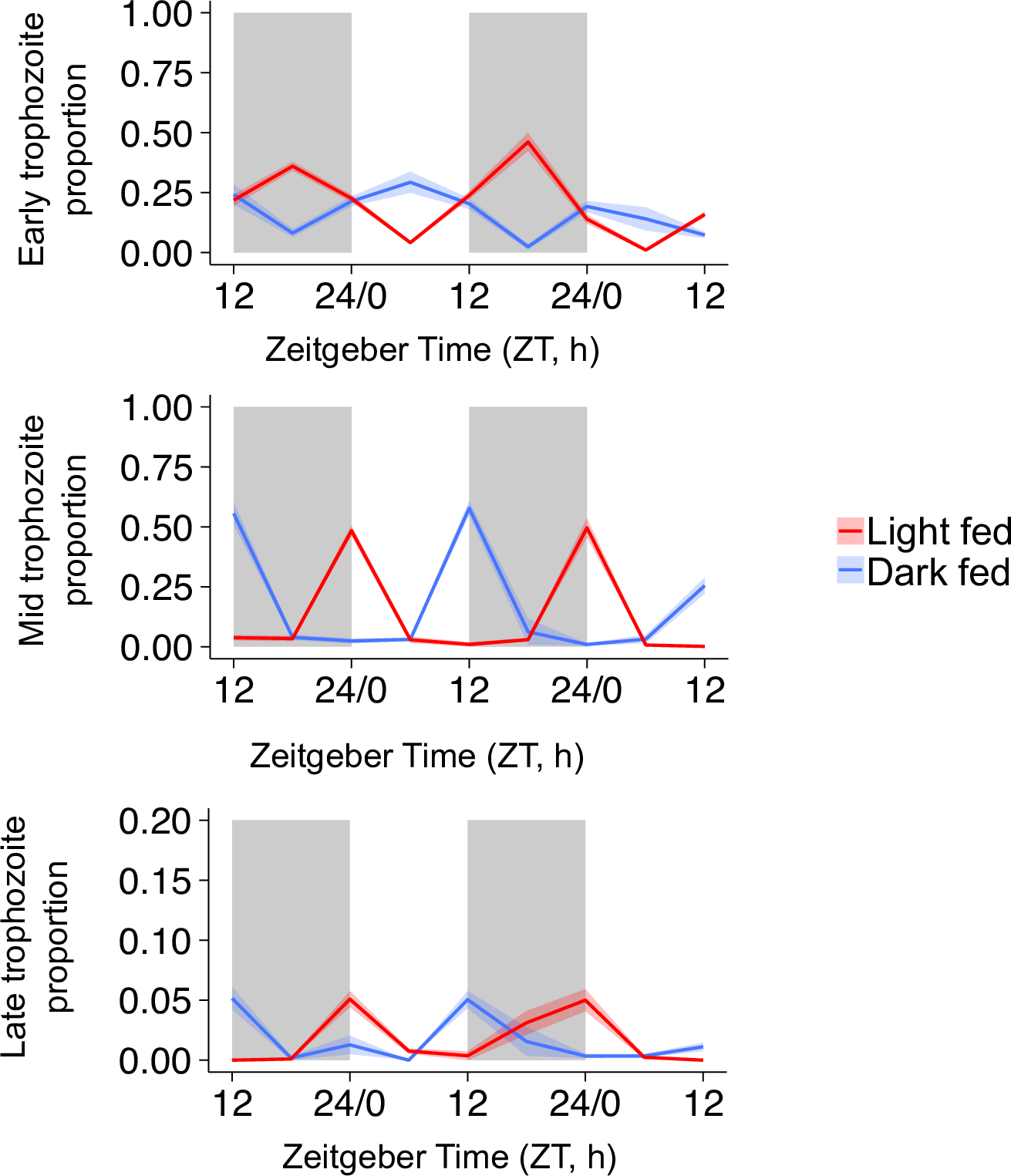

Supplement: S2 Fig — The proportion of parasites at early trophozoite, mid trophozoite and late trophozoite stages (ring stages are presented in Fig 3B) according to the feeding treatment of their hosts (light fed mice, red, and dark fed mice, blue). White and grey bars indicate lights on and lights off, respectively for both groups of mice (lights on ZT 0 and lights off ZT 12). Sampling occurred every 6 hours during days 6–8 post infection with means (± SEM, shaded area, N = 10) plotted for each treatment group. (TIF) [file ppat.1006900.s002.tif]

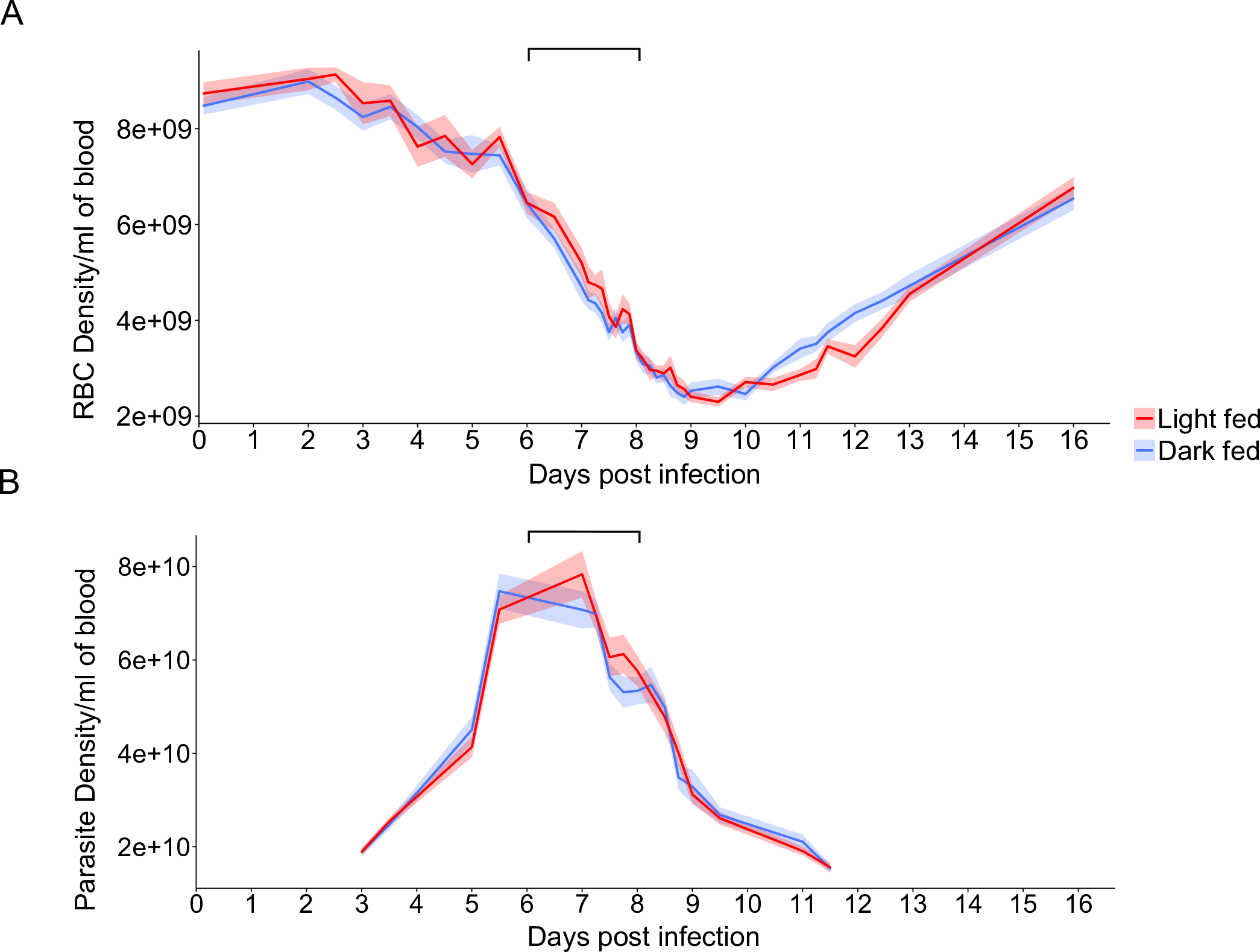

Supplement: S3 Fig — (A) Red blood cell density and (B) asexual parasite density for each treatment group (light fed mice, red, and dark fed mice, blue). Sampling occurred twice per day from days 0–5 and 9–16 post infection and 6 hourly during days 6–8 (indicated by bracket). Means (± SEM, shaded area, N = 10) plotted for each treatment group. (TIF) [file ppat.1006900.s003.tif]

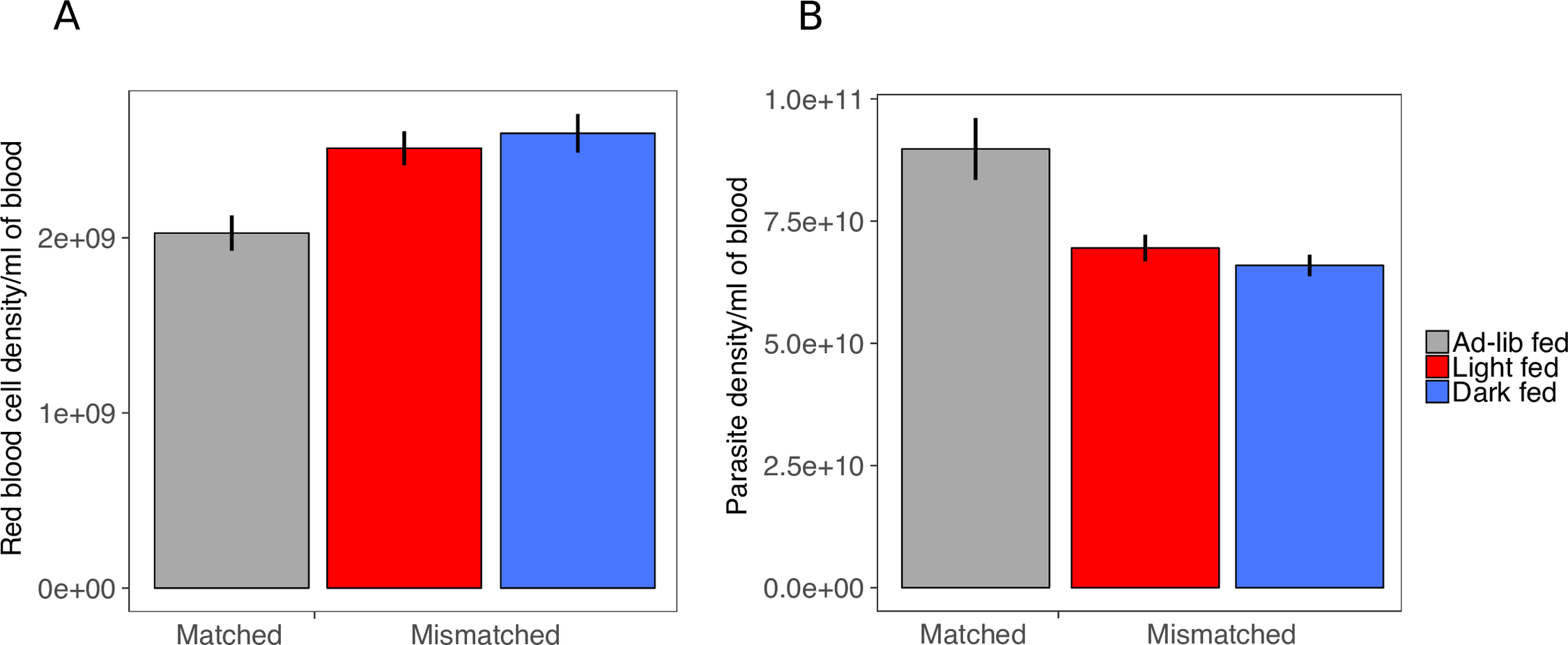

Supplement: S4 Fig — (A) Minimum red blood cell density and (B) parasite density. Sampling occurred on day 9 post infection for red blood cell density and day 6 post infection for parasite density. Means (± SEM, N≥12) is plotted for each treatment group (matched infections, grey; mismatched infections, light and dark fed are red and blue respectively). (TIF) [file ppat.1006900.s004.tif]

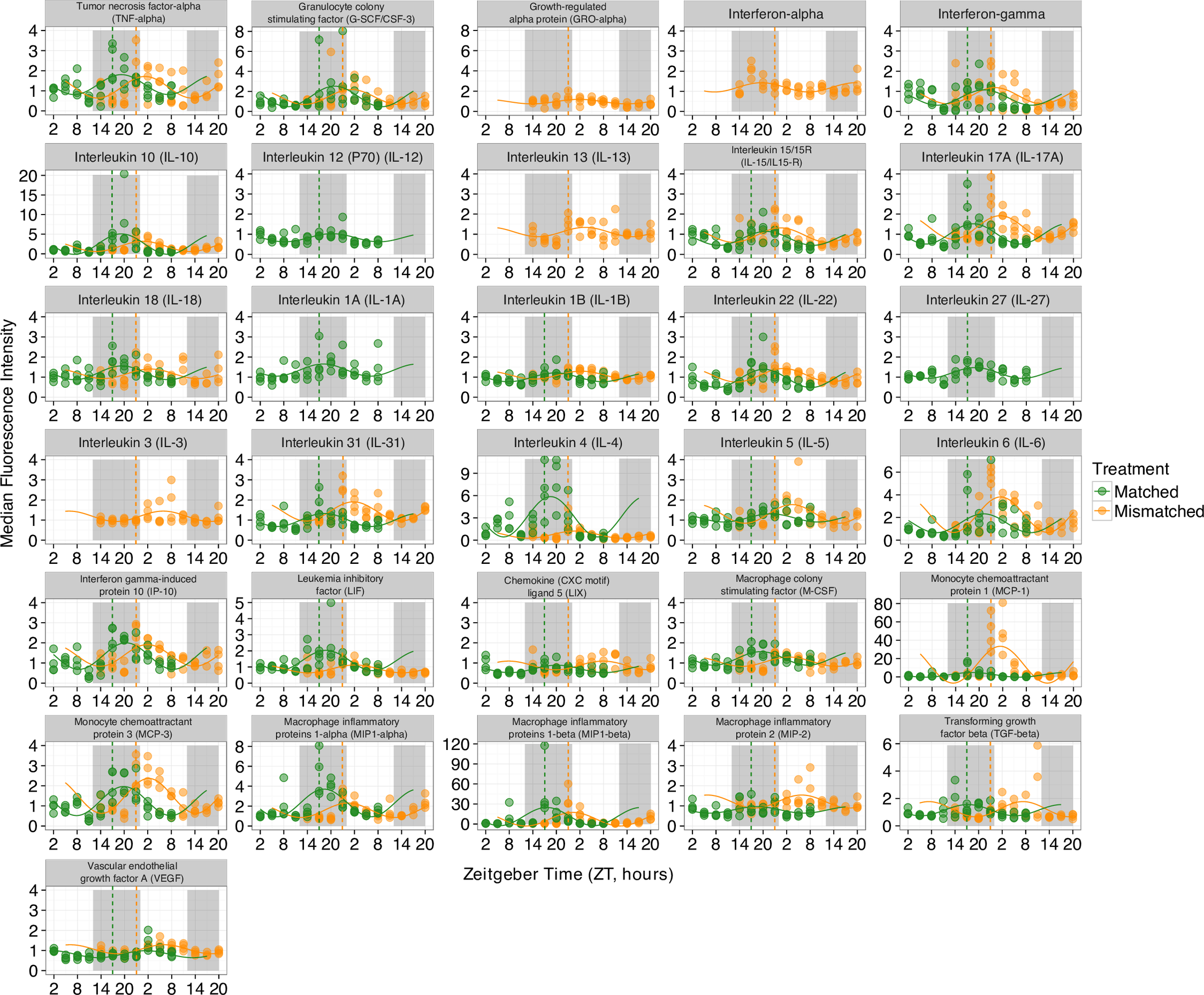

Supplement: S5 Fig — Median fluorescence intensity (used a proxy for cytokine concentration in pg/mol) for each infection and fitted curves for each treatment group (infections matched to the circadian rhythm of the host, green, and mismatched by 6 hours, orange). Sampling occurred every 3 hours on days 4–5 post infection. N = 4 per time point and parasites matched to the circadian rhythm of the host (green), undergo schizogony around ZT 17 (indicated by green dashed line) and parasites mismatched by 6 hours to the circadian rhythm of the host (orange), undergo schizogony around ZT 23 (indicated by orange dashed line). A panel of >40 cytokines and chemokines were assayed and all cytokines displayed are significantly circadian (estimated using CircWave). Non-significantly circadian cytokines include: both groups EOTAXIN, GM-CSF, RANTES, IL-2, IL-23, IL-9, IL-28; matched GRO-alpha, IFN-alpha, IL-13, IL-3; mismatched IL-12 (P70), IL-1 alpha, IL-27. Light and dark bars indicate lights on and lights off (lights on: ZT 0, lights off: ZT 12). (TIF) [file ppat.1006900.s005.tif]

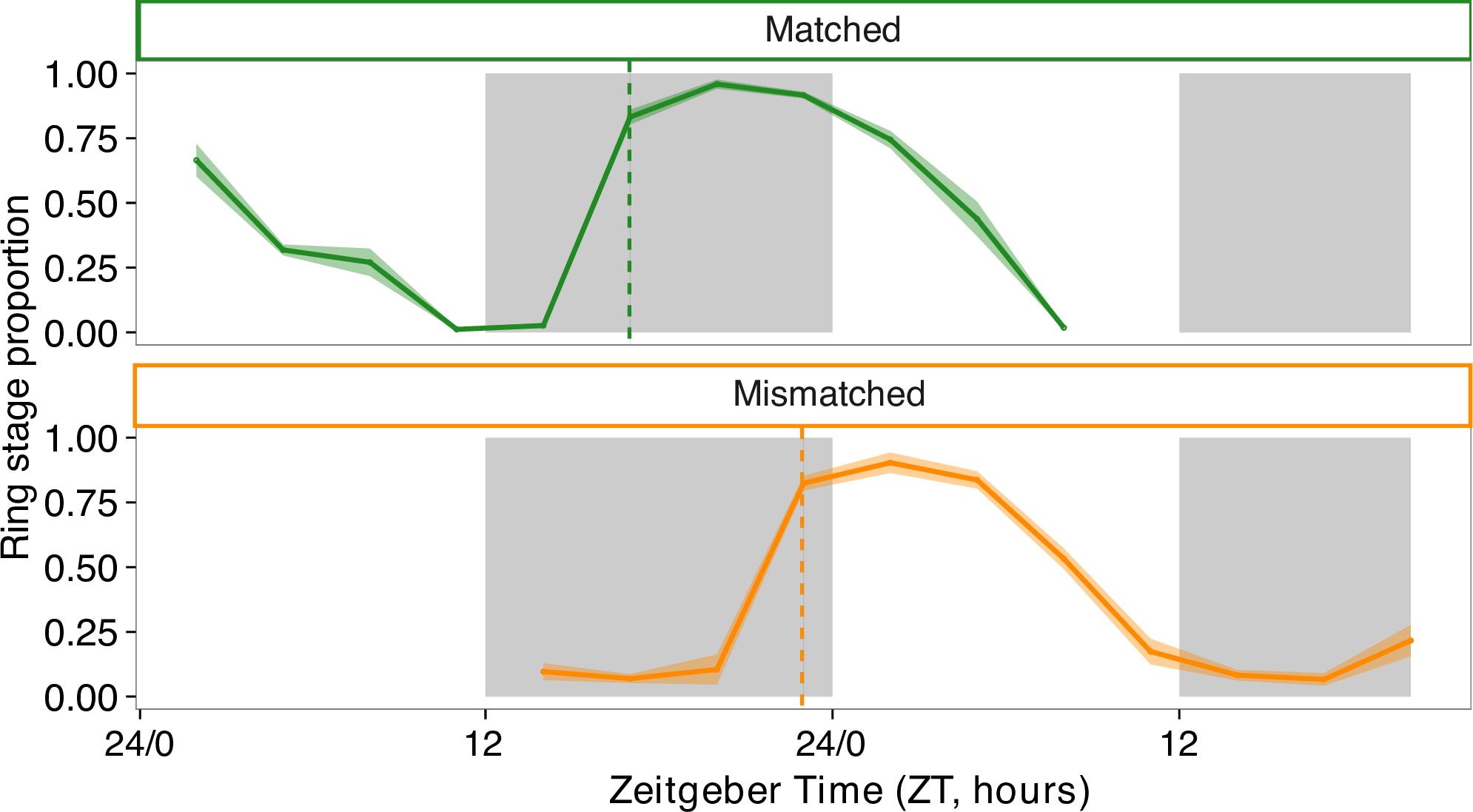

Supplement: S6 Fig — Mean ± SEM (N = 4 per time point) for parasites matched to the SCN rhythms of the host (green, undergo schizogony around ZT 17, indicated by green dashed line) and parasites mismatched by 6 hours to the circadian rhythm of the host (orange, undergo schizogony around ZT 23, indicated by orange dashed line). Analysis of rhythmicity for rings, and other, stages is presented in S4 Table. Sampling occurred every 3 hours on day 4–5 post infection. Light and dark bars indicate lights on and lights off (lights on: ZT 0, lights off: ZT 12). (TIF) [file ppat.1006900.s006.tif]
